# Identifying the tourism sector's exposure to climate change utilizing two different climate datasets: the case of three climatically diverse locations in Slovenia

**DOI:** 10.1007/s00484-025-02910-x

**Published:** 2025-05-01

**Authors:** Tjaša Pogačar, Rok Kuk, Katja Kokot, Maja Turnšek

**Affiliations:** 1 Biotechnical Faculty, University of Ljubljana, Jamnikarjeva 101, 1000 Ljubljana, Slovenia; 2 Ljubljana, Slovenia; 3 Faculty of Tourism, University of Maribor, Cesta Prvih Borcev 36, 8250 Brežice, Slovenia

**Keywords:** Climate change, Exposure, Tourism, Climate indicators, Data source, Uncertainties

## Abstract

This study investigates the exposure of Slovenia's tourism industry to climate change by analyzing climate data from two sources: the Copernicus Climate Data Store (CDS) and the Slovenian Environment Agency (ARSO). Three distinct climate zones in Slovenia, namely submediterranean, subcontinental and moderate climate of the hilly region are examined. Using climate indices such as *CIT: 3S* and *HCI: Urban*, the research assesses historical trends and future projections of climate suitability for various tourism activities. Key climate variables, including hot days, heavy precipitation, and snow cover, are analyzed to improve the understanding of climate exposure. The submediterranean region may experience extended tourist seasons but face challenges from heatwaves and water scarcity. The subalpine region, dependent on winter tourism, is projected to experience reduced snow cover and potential challenges for ski resorts. The subcontinental region could benefit from extended seasons for outdoor activities but may also face heat stress and extreme weather events. The study shows that climate indicators can offer valuable insights, but can also oversimplify complex climate processes. Discrepancies between CDS and ARSO data highlight potential biases, emphasizing the need for caution in interpreting absolute values. Climate projections inherently involve uncertainties, particularly for snow indicators. Ensemble modeling and careful consideration of uncertainties are essential for assessing future impacts. By addressing these considerations, this study provides a comprehensive understanding of climate change's implications for Slovenia's tourism sector and offers valuable guidance for adaptation planning.

## Introduction

Climate change is one of the most pressing global challenges of our time, with far-reaching implications for various sectors, including tourism. Data and climate change projections show that climate change will have a major impact on the tourism industry; however, the variability around the globe is high. According to the IPCC’s Sixth Assessment Report (IPCC 2022), the temperatures in Europe will continue to rise faster than the global mean, with largely negative impacts (e.g., increased cooling needs and water scarcity) projected for Mediterranean regions, including Slovenia. As temperatures rise, extreme weather events are becoming more frequent, posing significant challenges to the stability and sustainability of the tourism industry. Both snow-cover duration and snow depth in the Alps have decreased since the 1960 s, causing larger losses for the European tourism industry in Southern Europe (IPCC 2022). On the other hand, previous research has shown a projected prolongation of the seasons for outdoor tourism for three of Slovenia’s neighbouring countries: Croatia (Čavlek et al. 2019), Austria (Rudel et al. 2007), and Hungary (Kovács and Király 2021).

Tourism, being highly dependent on climate and weather conditions, faces both direct and indirect consequences of climate change. Giles and Perry (1998) discussed three major climate change impacts that would affect tourism: rising sea levels, higher temperatures and higher incidents of extreme weather events. They later added drought and rising sea temperatures as specific concerns for the Mediterranean (Perry 2000). That was also the time when UNWTO started to focus on climate change (Todd 2003; UNWTO 2003), leading to the Djerba and Davos Declarations (UNWTO 2004, 2007) and the first UNWTO Climate Change Report (Simpson et al. 2008). The report identified four key types of impacts of climate change on tourism destinations: direct impacts, such as geographic shifts in climate suitability for tourism and changes in operational costs; indirect environmental impacts, including water scarcity, biodiversity loss, landscape degradation, increased disease risks, and infrastructure damage; policy impacts, such as changes in tourist flows due to mitigation strategies; and indirect social impacts, including economic shifts, instability, and security concerns, affecting tourism operations and employment (Simpson et al. 2008). The latest climate change and tourism declaration, i.e. the Glasgow Declaration (UNWTO 2021), once again calls for the active involvement of tourism in mitigation efforts and focuses on its role in the»regeneration« of destinations as one of the responsibilities of tourism in relation to climate change adaptation. Although climate change adaptation has seen two decades of research in tourism, the most recent reviews still show important gaps (Scott and Gössling 2022; Remoaldo et al. 2024). The IPCC’s Sixth Assessment Report even showed a decline in the amount of research assessing climate change impacts on tourism compared to previous reports. According to Scott et al. (2023) the IPCC’s Sixth Assessment Report recognized tourism as an important stakeholder in adaptation efforts, yet it did not provide new insights into tourism adaptation compared to previous reports.

The vulnerability of a destination to climate change depends on its exposure to climate change, the sensitivity of the tourism system, and the adaptive capacity of the destination to cope with change (Čavlek et al. 2019). The intensity of tourism infrastructure development increases the level of exposure and vulnerability to climate change impacts, such as water scarcity, flooding and other climate events (eco-union 2019). Scott et al. (2019) proposed a Climate Change Vulnerability Index for Tourism (CVIT), comprised of 27 indicators to assess global tourism vulnerability to climate change. Three of these indicators are also relevant for the present study, which focuses on climate change exposure: Climate suitability for tourism, Ski tourism impact, and Weather disasters. Compared globally with the composite index of the 27 indicators, they assessed Slovenia’s tourism to be amongst the least vulnerable to climate change, next to most of the Global North. This comparison serves to stress the role of affluent countries in advocating for a global just transition, including the tourism sector (Bigby et al. 2024). The global comparison, however, does not aid in providing specific recommendations on minimizing the risks and maximizing the opportunities that Slovenia’s tourism sector should focus on while adapting to climate change.

Many adaptation options do not only address climate change risks but also a response to a broader set of climatic (e.g., extreme weather events, water scarcity) and non-climatic factors (e.g., general diversification of markets, general commitment to sustainability). Adapting to the impacts of climate change on tourism is imperative not only for the industry's survival but also for the well-being of communities dependent on tourism-related activities. However, according to the first results of the Global Survey on Climate Action in Tourism (Pilgreen 2022), the adaptive capacity amongst approximately 1100 survey respondents is largely lacking, and tourism organisations do not routinely review their climate objectives or assess present and future risks. It is, therefore, an important role of climatologists to prepare an assessment of exposure and stress the severity of risks to tourism stakeholders.

Understanding the complex relationship between climate change and tourism is crucial for formulating effective policies and strategies to foster resilience in the face of a changing climate. The effects of climate change on tourism are often described using climate indicators that are adapted to the specific weather requirements for a particular type of tourism. One of the first indices was the tourism climatic index (*TCI*) proposed by Mieczkowski (1985). It was designed as a composite measure of the well-being of tourists, letting them choose the time of the year when climate conditions are at their optimum or the area that offers the most suitable climate conditions during their fixed holiday period. De Freitas et al. (2008) developed a second-generation climate index, named the climate index for tourism (*CIT*), designed initially to rate beach tourism (*3S*: sun, sea and sand). *CIT* was later adapted to specifically appraise cycling, cultural tourism, football, golf, motor boating, sailing and hiking with a demonstration of the suitability of the future climate for outdoor activities for the Bay of Palma on the island of Majorca (Bafaluy et al. 2014). Even then, research showed that future summers would be too hot for many outdoor activities. The holiday climate index (*HCI)* was developed by Tang (2013) and later upgraded by Scott et al. (2016) to overcome the deficiencies of the *TCI*. Further, the *HCI: Urban* was explicitly designed for sightseeing and other general outdoor activities of leisure tourists in urban destinations. To overcome deficiencies of the *TCI*, the *HCI* variable rating scales and the component weighting system are based on the available literature (theoretically sound) and a range of surveys over a decade (empirically tested); they include all facets of climate and are simple to calculate, and easy to use and understand (Tang 2013; Scott et al. 2016).

The escalating impacts of climate change have underscored the urgent need for accessible and reliable climate data. While an information system capable of transparently displaying both global and regional climate data is essential (Thepaut and Dee 2016), the proliferation of smaller, inconsistent databases can hinder data interpretation (Merks et al. 2020). To address these challenges, the European Center for Medium-Range Weather Forecasts (ECMWF) partnered with the European Commission in 2014 to establish the Copernicus Climate Change Service (C3S) (Raoult et al. 2017). The Climate Data Store (CDS) is a cornerstone of C3S, providing a centralized platform for accessing, analyzing, and preparing diverse climate data for monitoring and projection purposes. Rigorous quality control and data homogenization processes ensure data reliability within the CDS. However, the question is whether it is possible to use this kind of climate change evidence to build a bridge between academic research and practical climate risk assessment policies (Arabadzhyan et al. 2021).

The purpose of our research was to comprehensively analyse the exposure of the tourism sector in Slovenia to climate change, assess the sensitivity of various destinations and types of tourism, and provide relevant recommendations for climate change adaptation measures for tourism stakeholders. In this paper, we present one part of the research with an emphasis on the use of different climatological data on the local level with appropriate uncertainty explanations to help the tourism sector understand possible future implications of climate change.

## Materials and methods

**Fig. 1:**
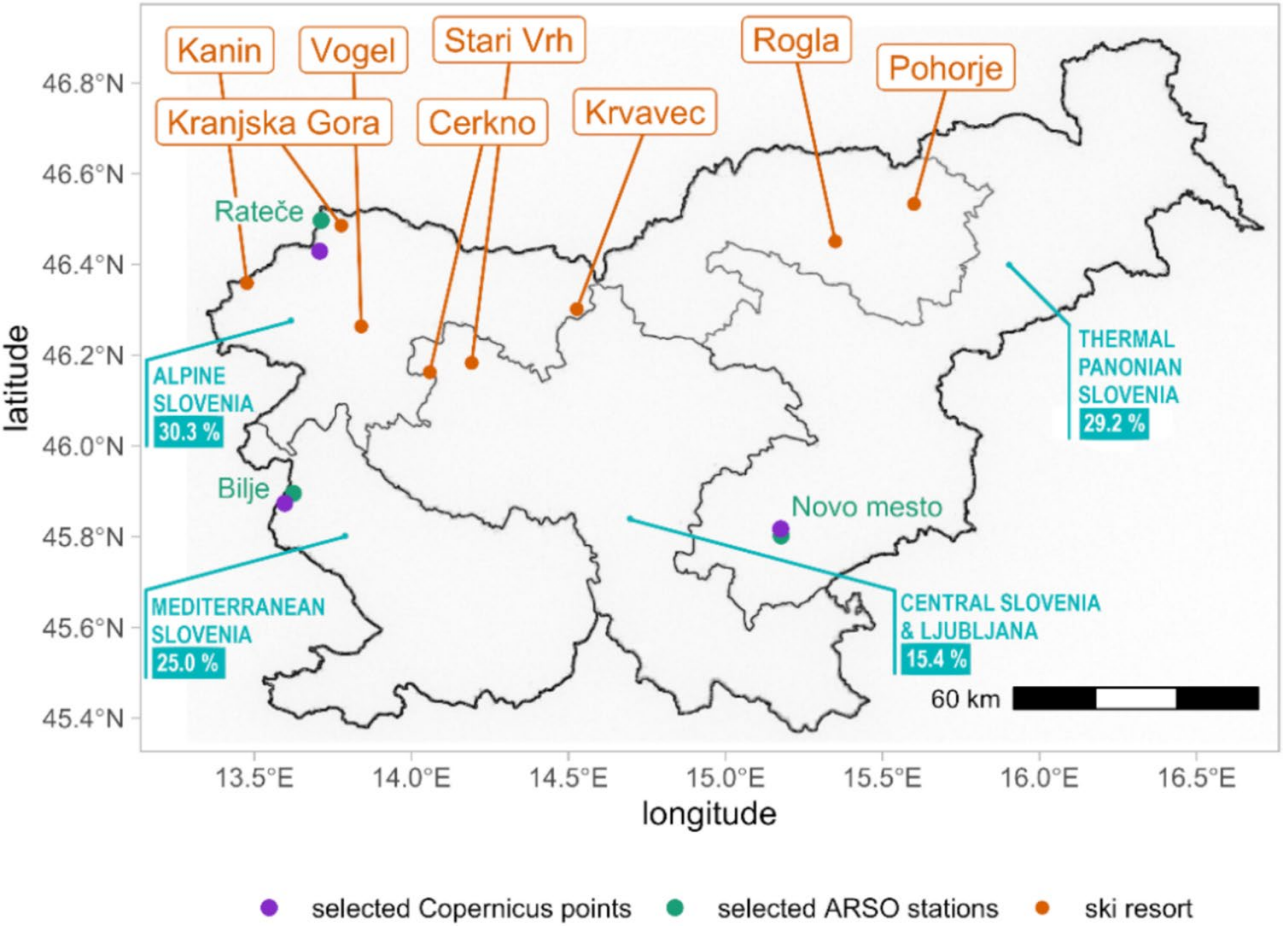
The map of Slovenian tourism macro-destinations (blue names with % of all overnight stays in Slovenia), ski resorts (orange names) and selected ARSO stations and Copernicus points (adapted from Turnšek et al. 2024 and STO 2017)

In Slovenia, there are three European climate zones that define the tourist offer: the Mediterranean, the Alps, and the Pannonian. The hereby presented analysis of climate change impacts and future hazards for Slovenian tourism is based on determining the tourism activities selected for the analysis. This selection then guides the selection of climate data, such as the CIT and HCI, and snow cover duration. The variables or indices were selected for each area based on the leading tourism products identified by the two last national tourism strategies (STO 2017, 2022). Based on the different strategic focus on tourism activities, the national tourism strategy (STO 2017) identified four different so-called macro-destinations, which, to some degree, correspond with the climatic areas of the country (Fig. 1).

Mediterranean macro-region is characterised by a submediterranean climate (example: Bilje) with dry and hot summers and mild winters in the southwestern part of Slovenia. The leading tourism products in this climate are summer outdoor tourism, urban and cultural tourism, MICE tourism (business meetings & events), culture, touring (for urban activities) and sun & sea tourism by the Slovenian coast. Ljubljana and central Slovenia and Thermal Pannonian Slovenia are mainly characterised by subcontinental climate (example: Novo mesto) with relatively cold winters and hot summers and large amounts of precipitation, especially in the mountains; the exception is the northeastern part (the Pannonian plane), which is the driest. These areas focus on summer outdoor tourism, urban and cultural tourism, MICE tourism, and thermal spas in the east. The moderate climate of hilly regions is typical for a large (hilly and not yet mountainous) part of Alpine Slovenia (example: Rateče), where the main tourism products are winter outdoor tourism and summer outdoor tourism.

The study utilizes climate data and projections from two primary sources: the Copernicus Climate Data Store (CDS) and the Slovenian Environment Agency (ARSO). Future climate projections are based on Representative Concentration Pathways (RCPs), which outline different greenhouse gas emission scenarios. We use three RCPs: RCP2.6 (optimistic), RCP4.5 (intermediate), and RCP8.5 (pessimistic). To account for uncertainties in climate modeling, we employ multiple models and present results as a median with a range. Projections indicate the direction and magnitude of changes in climate variables rather than providing absolute future values. This approach reflects the inherent limitations and variability among climate models. Climate projections of air temperature and the resulting indicators, such as hot days and tropical nights, are much less uncertain than simulations of precipitation, where natural variability in space and time is much greater. The reliability of projections of relative humidity, cloud cover, and the like, which are also used to determine tourism climate indicators, is even lower.

### Climate indices for tourism

To assess the potential impacts of climate change on tourism in Slovenia, we employed two climate indices: *CIT: 3S* (Climate index for tourism: sun, sea and sand; De Freitas et al. 2008) and *HCI: Urban* (Holiday Climate Index; Scott et al. 2016). These indices are based on tourist climatic preferences, providing a more objective and relevant assessment of climate suitability for beach and urban destinations (Cardell et al. 2022). Recent studies have demonstrated the applicability and value of these indices in various contexts. For instance, HCI has been successfully applied to assess tourism climate in Italy (Mazzarano et al. 2024) and the Mediterranean Coast of Turkey (Bilgin et al. 2024), while CIT has been used to evaluate climate suitability for tourism in Spain (Cardell et al. 2022). Furthermore, the availability of CIT and HCI within the Copernicus CDS (Benassi et al. 2019) offered significant advantages. Their European-wide coverage promotes greater accessibility, facilitates comparative analyses across different regions, and encourages wider adoption within the tourism sector. Unfortunately, during the process of publishing the paper, this CDS dataset became unavailable for no reason except to establish a new system, which means methods may not be replicable in this part (using CIT and HCI scores projections from the CDS).

*CIT: 3S* (de Freitas et al. 2008) evaluates climate suitability for tourism based on thermal sensation (*TS*), aesthetic appeal (*AA*), and physical conditions (*PC*). The thermal sensation is like in Morgan et al. (2000) determined by skin temperature (*T*_*s*_ [°C]), following Green (1967):

$$T_s=T_M+\frac17hM+\frac{M-15+120s(1-A)}{2+9\cdot\sqrt{0.1+W}},$$

where ${T}_{M}\; [^\circ C]$ is the maximum daily temperature, *s* [%] is a reciprocal of total cloud cover, *W* [m/s] is the wind speed; and standardized are the thickness of clothing (*h* = *0.008 cm*), the metabolic rate (*M* = *25 cal/s*), and the albedo of clothing and skin (*A* = *0.45*) (same as in Morgan et al. 2000).

Aesthetic appeal considers cloud cover, and physical conditions encompass wind strength and precipitation levels. The index categorizes climate comfort into seven levels, from very poor (0–3), through marginal (4) to ideal (5–7), using the matrix of these components to determine the level (Table 1). The equation is *CIT* = f [TS, AA)*PC] and the most limiting condition has to be used (adapted from de Freitas et al. 2008).

**Table 1:** The matrix for *CIT: 3S* (beach tourism) (adapted from de Freitas et al. 2008 and Morgan et al. 2000 (*T*_*s*_))

Skin temperature T_s_ [°C]	ASHRAE scale [TS]	Cloud (≤ 50%) [AA]	Cloud (> 50%) [AA]	Rain (> 3 mm/day) [PC]	Wind (≥ 6 m/s) [PC]
> 35.5	Very hot	4	3	2	3
34.5–35.5	Hot	6	5	2	4
33.5–34.5	Warm	7	5	2	4
32.5–33.5	Slightly warm	6	4	1	4
31.0–32.5	Indifferent	5	3	1	2
29.0–31.0	Slightly cool	4	3	1	2
26.0–29.0	Cool	0	0	0	0
21.0–26.0	Cold	0	0	0	0
< 21.0	Very cold	0	0	0	0

On the other hand, *HCI: Urban* combines thermal comfort (*TC*), which is in Benassi et al. (2019) calculated as effective temperature (*ET [°C]*), derived by Missenard's formula (*e.g.* Gregorczuk and Cena 1967) from maximum temperature (*T [°C]*) and minimum humidity (*RH* [%]):

$$ET = T - 0.4(T - 10) + (1-RH/100),$$

with aesthetic aspects (*A* − cloud cover) and physical conditions (*P* − precipitation and *W* − wind). These components (see Table 2 to determine scores from –10 to 10) are weighted and summed to produce an index ranging from 0 to 100, categorized as ideal (*HCI* > *70*), marginal (*50* < *HCI* < *70*), or very poor (*HCI* < *50*) conditions (Scott et al. 2016):

$$HCI=4\left(TC\right)+2A+\left(3\left(P\right)+W\right)$$

**Table 2:**
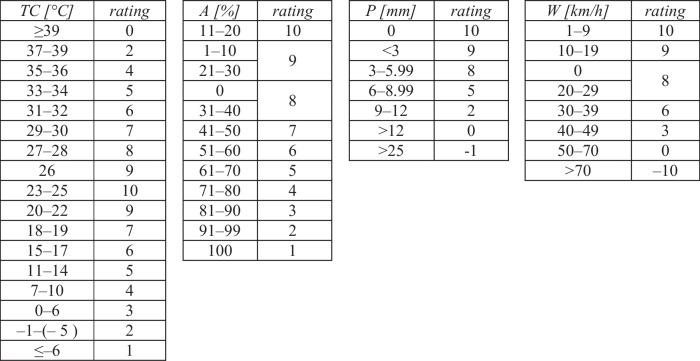
Rating system for the components of *HCI: Urban: TC* – thermal comfort from effective temperature, *A* – cloud cover, *P* – precipitation, *W* – wind (adapted from Scott et al. 2016)

Using the CDS dataset"Climate suitability indicators for tourism from 1970 to 2100 over Europe derived from climate projections"(CDS 2022), we analyzed both historical (1986–2005) and projected (2021–2040, 2041–2060, 2081–2100) climate data for the selected indices. Based on EURO-CORDEX projections and a 0.11° grid resolution, the dataset provides monthly counts of days within each climate comfort category. To account for model variability, we averaged results across six models and used error bars to represent the 10 th and 90 th percentiles. We selected the points on the grid closest to major tourist destinations in Slovenia. We also ensured the selected points were near weather stations operated by ARSO to compare the historical data from these weather stations to the historical data in CDS (three points presented in this paper: Rateče, Bilje, Novo mesto).

For validation and comparison, we calculated *CIT: 3S* and *HCI: Urban* values using historical ARSO weather station data for the periods 1971–2000, 1981–2010, 1991–2020 (usually used in Slovenia), and 1986–2005 (used in CDS). These calculations were based on maximum temperature, wind speed, cloud cover, precipitation, and relative humidity, employing the same methodology as CDS. However, due to data limitations, ARSO projections were unavailable, preventing direct comparison of projected indices between the two datasets.

### Other climate variables important for tourism

We analysed climate data and projections provided by ARSO (2022) for six variables: number of days with at least 1 mm of precipitation, number of days with at least 20 mm of precipitation, number of days with snow cover, number of tropical nights (T_min_ ≥ 20 °C); number of warm days (T_max_ ≥ 25 °C); and number of hot days (T_max_ ≥ 30 °C), where T_min_ is minimum daily temperature and T_max_ is maximum daily temperature. The projections are based on averages of simulations of different regional climate models, six for the RCP4.5 and RCP8.5 scenarios and two for the RCP2.6 scenario, which are based on historical data best simulated the climatic conditions in Slovenia. Bias correction has been performed for temperature and precipitation projections. ARSO data was provided for four time periods (2011–2040, 2041–2070, 2071–2100 vs. the reference period 1981–2010) and three RCPs.

### Snow indicators from CDS

*CIT: 3S* and *HCI: Urban* were not prioritized in the Subalpine climate zone. Although these indices were analyzed within a broader research context, their relevance to the specific research questions of this paper was limited. Our primary focus in this region was on factors directly impacting winter tourism, specifically snow conditions and the feasibility of artificial snow production.

A relatively new Ski Climate Index (SCI; Demiroglu et al. 2021) offers a promising approach to assessing ski resort suitability by combining snow reliability and meteorological conditions. However, its application in Slovenia is hindered by limited high-altitude data and unreliable precipitation projections, which could lead to inaccurate conclusions. Therefore, striving for European-wide coverage and standardized methodology for the tourism sector, which facilitate further comparative analyses across different regions and promote broader adoption, we utilized the number of days with at least 5 cm of natural snow cover (August 1 st to July 31 st) and potential snowmaking hours (wet bulb temperature below − 2 °C) in November and December. Both indices are part of the"Mountain tourism meteorological and snow indicators for Europe for tourism from 1950 to 2100 derived from reanalysis and climate projections"dataset available in CDS (CDS snow 2022). This dataset incorporates climate variables derived from reanalysis data and EURO-CORDEX models. Climate model outputs were aggregated over 20-year periods, and quantiles of annual values were calculated for each RCP and period. The dataset's methodology is detailed in Morin (2020).

The data is presented at the NUTS- 3 regional level with a 100 m elevation resolution. Importantly, each data point represents a specific location within a region, rather than an average. To generate the dataset, locations were selected within each region and elevation band. Consequently, data points at adjacent elevations might be geographically distant. In the main research, we selected eight major Slovenian ski resorts (Fig. 1; orange names) and categorized them into three NUTS- 3 regions. In the paper, the emphasis is on the location of Rateče near the ski area Kranjska Gora. It's crucial to note that while this analysis provides valuable insights into climate trends at specific elevations within regions, direct comparisons between different elevations within a region should be interpreted cautiously due to potential geographical disparities. For each ski resort, we chose two elevations, one at the lowest part of the ski resort and another at the highest part of the ski resort or near the top, based on the availability of data for those elevations. The spread is indicated with error bars, which show the 10 th and 90 th percentile of values across all models.

## Results

### An example of impacts in the submediterranean climate

**Fig. 2:**
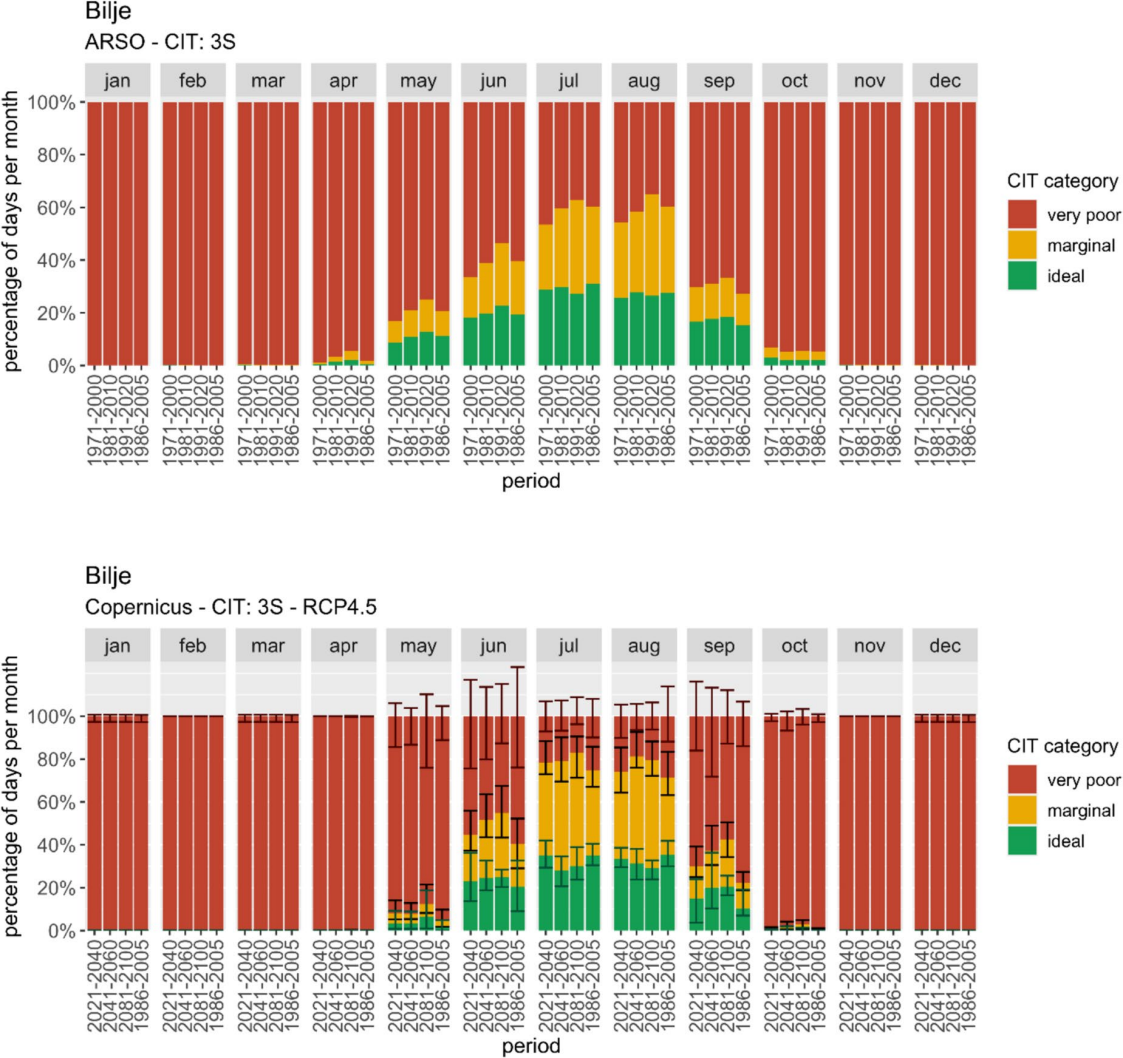
Monthly percentage of ideal/marginal/very poor days according to *CIT: 3S*. Top: Calculated using ARSO data for past periods (the last one to compare with CDS); bottom: representation of CDS data and projections using RCP4.5 scenario

Bilje (altitude 55 m) is a meteorological station in the Karst Mediterranean region of Slovenia, 80 km from the seaside. Analysis of the *CIT: 3S* index reveals a lengthening of the suitable season for 3S tourism, primarily extending into spring (Fig. 2).

Comparisons between CDS and ARSO datasets highlight discrepancies, emphasizing the importance of cautious interpretation of absolute values. The difference in the average percentage of ideal days in September in 1986–2005 is almost 5%, and in May, even 10%, CDS underestimates the ideal conditions; the difference during the summer is not so noticeable. Projections suggest a stable percentage of days with ideal climate comfort for water-based tourism during summer but a notable increase in spring and autumn, potentially extending the tourism season. However, a decline in ideal conditions is projected for July and August, especially under the RCP8.5 scenario (not shown).

**Fig. 3:**
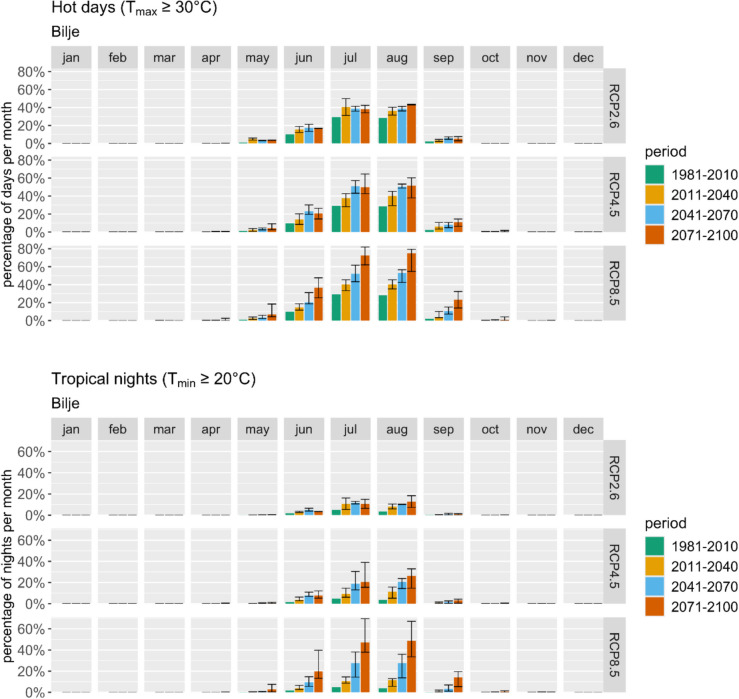
Average monthly percentage of hot days (top) and tropical nights (bottom) represented using ARSO data and projections for the reference period and three future periods and scenarios

Projected climate changes in Bilje indicate a substantial increase in temperature, particularly the frequency of warm and hot days (Fig. 3 top), with the uncertainty being much higher for hot than for warm days. The range of the projected changes is presented with whiskers, which show the range from the minimum to the maximum modelled change that should be analysed in the form of deviations and not absolute values. To be exact: for RCP8.5 projections for July show a definite increase in the number of hot days in Bilje in the first period (2011–2040) by 3 days compared to the reference period (1981–2010), with the possible range from 1 to 5 days, in the second period (2041–2070) by 7 days, with the range from 5 to 10 days, and in the third period (2071–2100) by 13 days, with the range from 10 to 16 days. However, for a clearer general picture, we show graphs with the absolute number of days that meet or will meet a certain criterion; however, the interpretation of the graphs is essential. Median values that are mainly described have to be taken with caution, bearing in mind the whole variability and reliability. Additionally, the occurrence of tropical nights (Fig. 3 bottom) is essential to assess, causing unsuitable conditions for the human body to rest if inside temperatures are not lower or for tourists staying outside. Projections show a substantial increase towards the end of the century, much worse with each scenario.

Regarding precipitation (not shown), a decrease in days with at least 1 mm of precipitation is anticipated for the summer months, particularly under the RCP8.5 scenario. Especially within the RCP8.5, the reliability of projections for spring and early summer is not very high. However, other scenarios show that for July and August and partly also September, the number of days with at least 1 mm of precipitation is projected to decrease, while winter months show a slight increase. Summer is thus projected to continue to be mostly dry, while the projections for the shoulder seasons show variable results and, thus, not a very clear trend. Conversely, an increase in heavy precipitation events is projected for June, September, and October, emphasizing the potential for more intense rainfall during these periods.

### An example of impacts in subcontinental climate

**Fig. 4:**
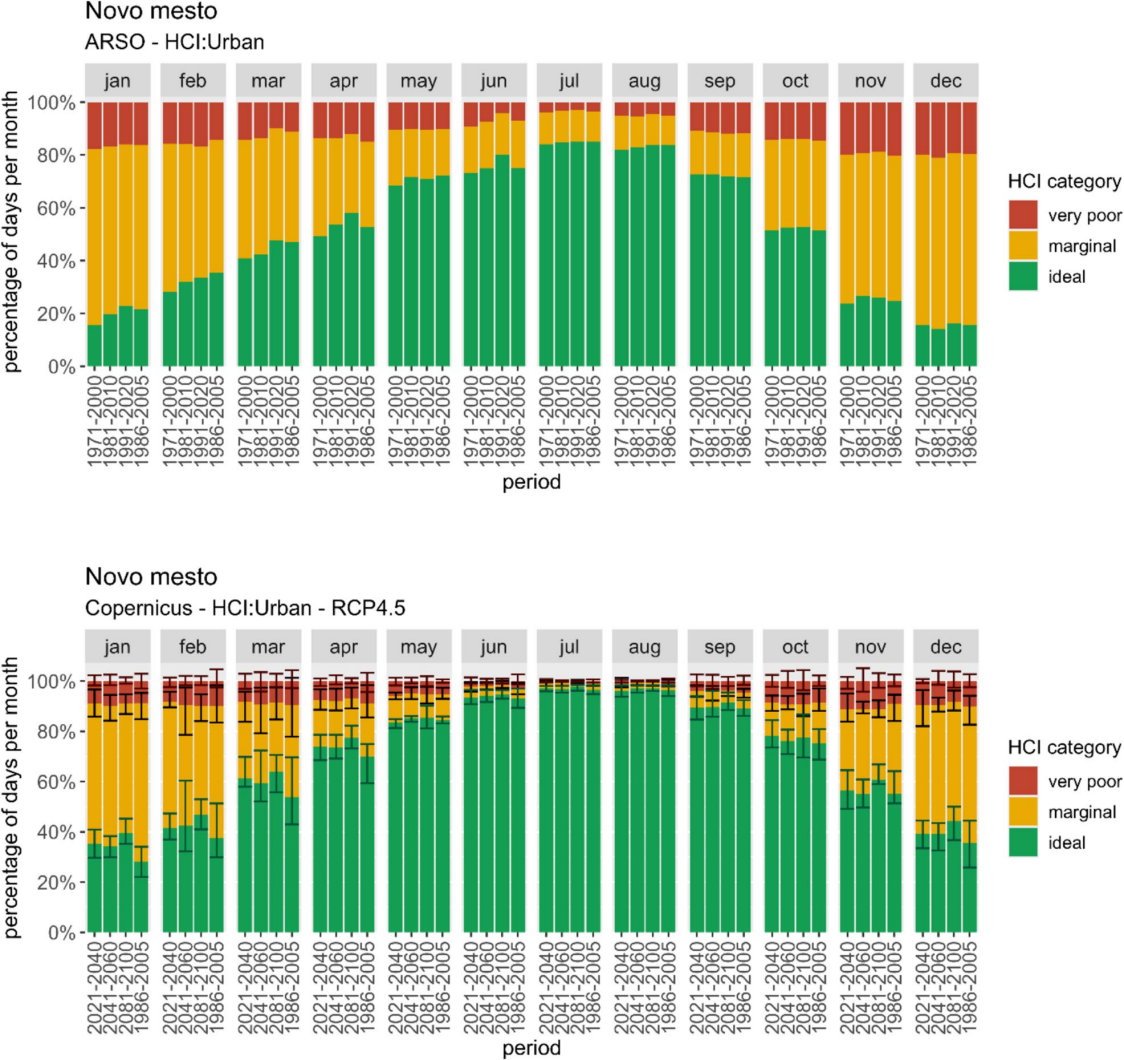
Monthly percentage of ideal/marginal/very poor days according to *HCI: Urban*. Top: Calculated using ARSO data for past periods (the last one to compare with CDS); bottom: representation of CDS data and projections using RCP4.5 scenario

For Novo Mesto, summer can be highlighted as the peak season for ideal outdoor health tourism conditions. Novo Mesto and its surroundings are a popular tourism destination in Slovenia, largely due to its well-known health resorts (Rajver et al. 2020). The CIT:3S index was used for interpretation as beach and outdoor health tourism need similar favourable weather conditions to ensure optimal activity experiences. Notably, this suitable period has been extending into the spring months. Projections of *CIT: 3S* (not shown) indicate a future increase in days with marginal climate comfort for tourism during summer, offset by a rise in ideal conditions during September and spring. *HCI: Urban* for Novo Mesto reveals favorable conditions for urban tourism throughout the year. Past data (Fig. 4 top) indicates that the summer months (July and August) experienced the highest percentage of days with ideal climate comfort, reaching approximately 80%. However, the comfort season has extended into early spring, and even winter months have boasted over 80% of days with marginal or ideal conditions. Projected data (Fig. 4 bottom) suggests a continuation of favorable summer and late spring conditions. Notably, autumn, winter, and early spring are anticipated to experience an increase in days with ideal climate comfort. While winter previously accounted for around 20% of days with very poor climate comfort, a slight decrease is expected. Summer conditions remain exceptionally favorable for urban tourism, promising excellent climate conditions year-round.

The differences between ARSO and CDS data for the period 1986–2005 are the most obvious in this case and should be considered when representing CDS projections. For July and August, CDS data show almost only ideal days, while local ARSO data show an average of 80%. CDS is highly overestimating the percentage of ideal days also in other months in the range, comparable to the impact of projected climate change.

**Fig. 5:**
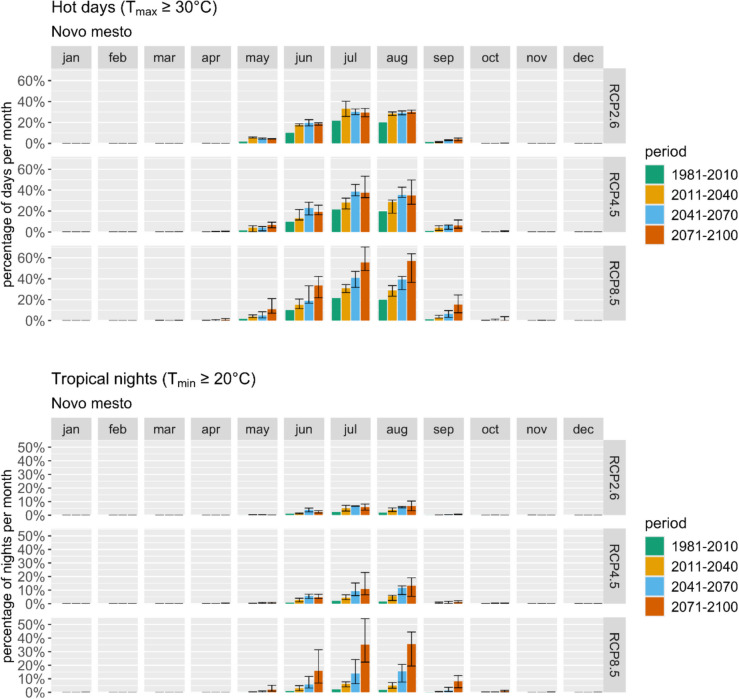
Average monthly percentage of hot days (top) and tropical nights (bottom) represented using ARSO data and projections for the reference period and three future periods and scenarios

Analysis of the average effective temperature itself (as a part of climate indices calculations) in Novo Mesto reveals a steady increase throughout the periods. While this has improved thermal comfort in May, it has worsened conditions in June, July, and August. Projections for warm and hot days (Fig. 5 top) indicate a significant temperature rise, particularly during the summer months. The RCP8.5 scenario presents the most severe outlook, with a potential doubling or tripling of hot days in July and August. Despite high variability, the overall trend of increased hot days is certain. Similarly, there is a noticeable increase in the number of tropical nights projected (Fig. 5 bottom), especially high according to RCP8.5 for the late century.

From the analysis of these climate variables, it's evident that heat stress will increase during summer, although it can not be seen from *HCI: Urban*, which mainly shows ideal conditions, masking the negative impacts of climate change. To maintain favorable urban comfort, the implementation of adaptation measures, especially for the summer months, is crucial.

**Fig. 6:**
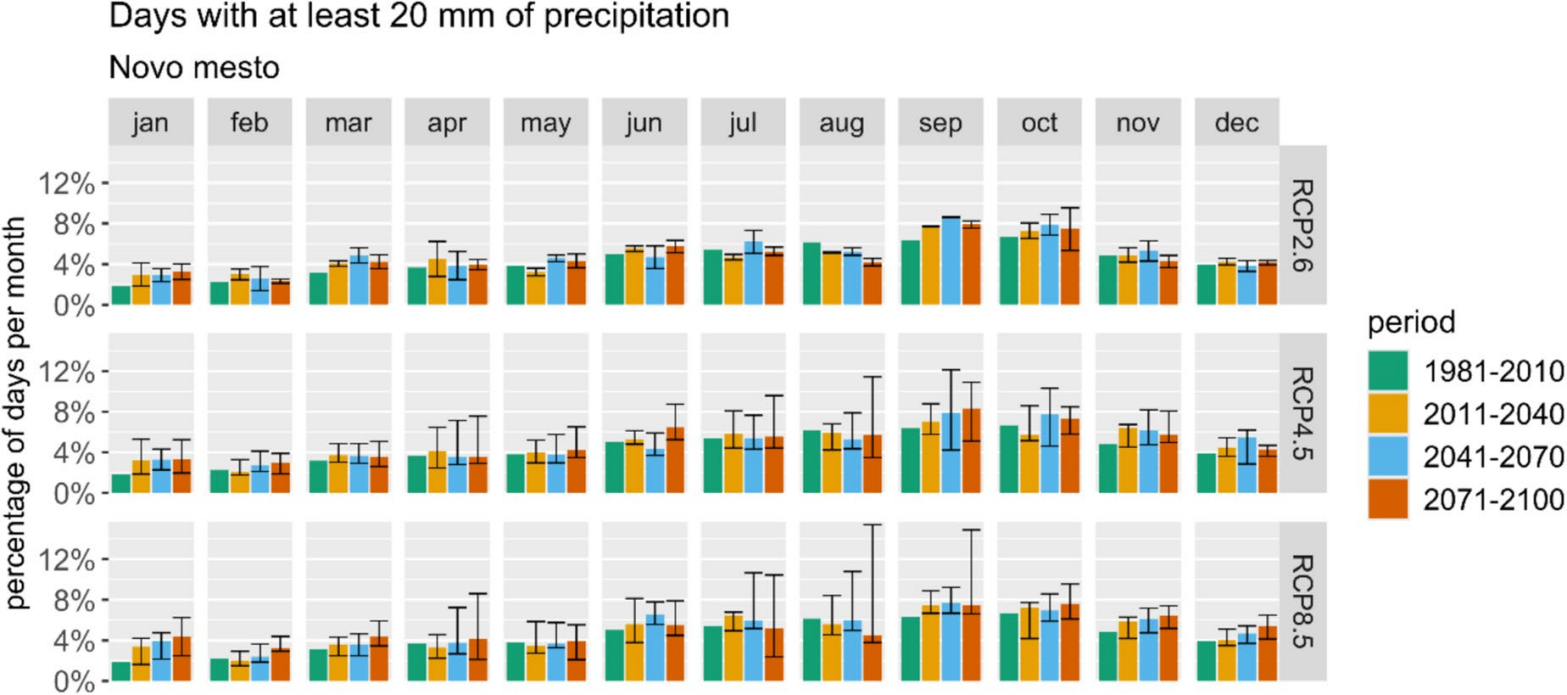
Average monthly percentage of days with at least 20 mm of precipitation represented using ARSO data and projections for the reference period and three future periods and scenarios

Figure 6 presents projections of days with at least 20 mm of precipitation for Novo Mesto across three scenarios. While all scenarios indicate a rise in heavy precipitation events during autumn and winter, the primary concern for tourism lies in summer conditions. A potential decrease in August could mean safer conditions for outdoor tourism.

### An example of impacts in subalpine climate

**Fig. 7:**
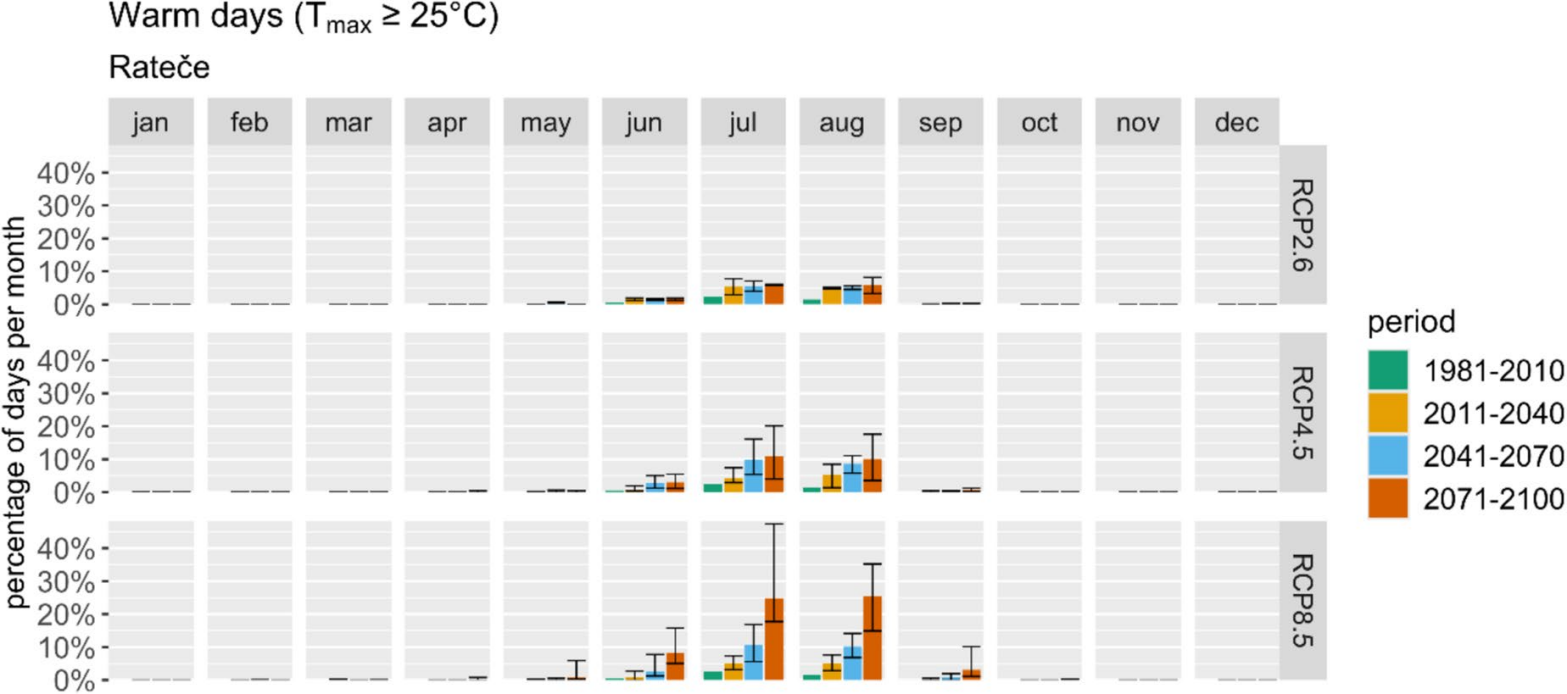
Average monthly number of warm days represented using ARSO data and projections for the reference period and three future periods and scenarios

Rateče, situated within the Julian Alps, is poised to experience significant climatic shifts with profound implications for its tourism industry. Projections indicate a notable increase in warm days (Fig. 7), particularly under the RCP8.5 scenario, suggesting potential growth in summer tourism.

**Fig. 8:**
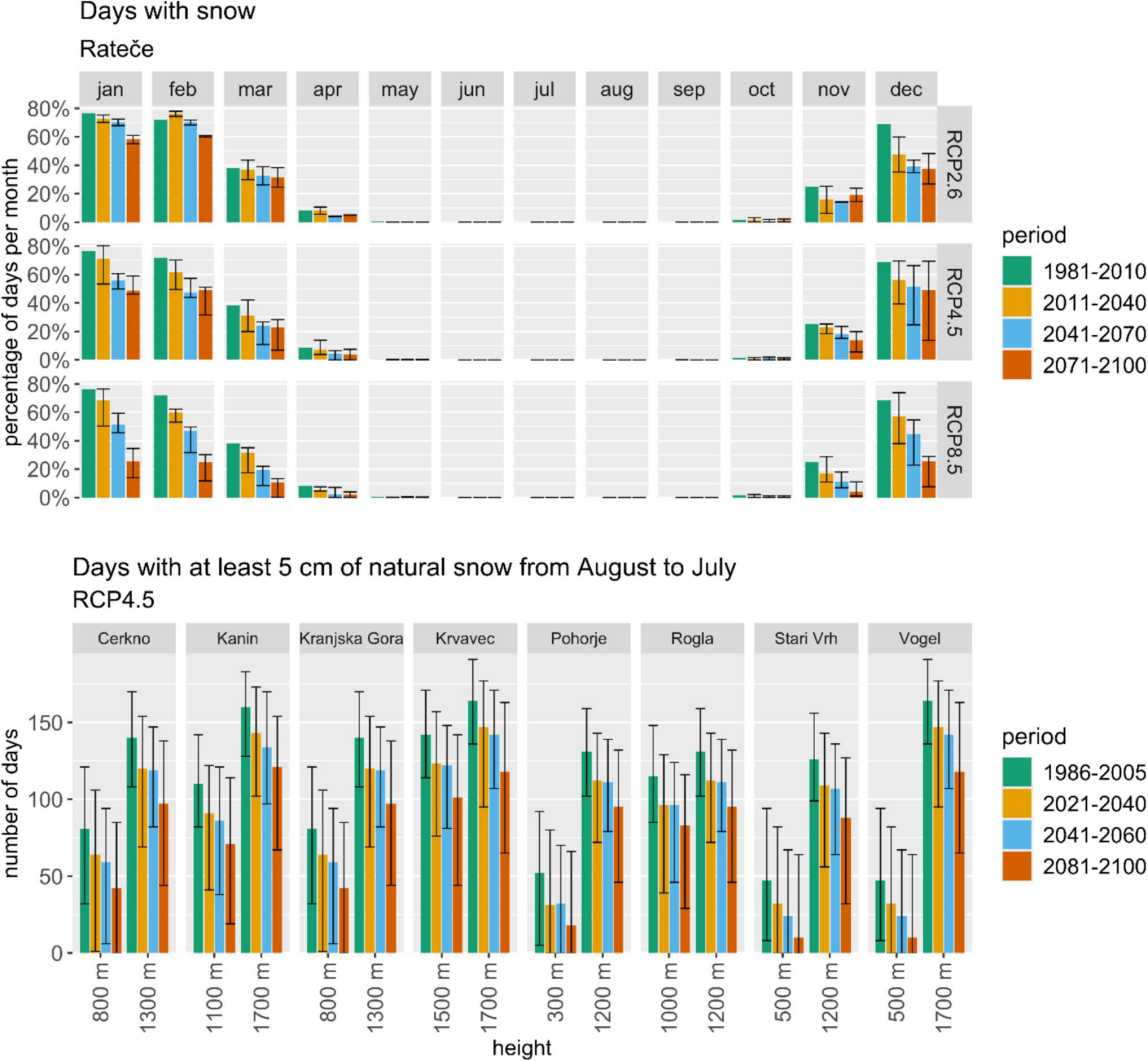
Top: Average monthly number of days with snow represented using ARSO data and projections for the reference period and three future periods and scenarios in Rateče. Bottom: Representation of CDS data and projections of the average seasonal number of days with at least 5 cm of natural snow for various ski areas in Slovenia at two heights for the reference period and three future periods and scenarios

However, the region's dependence on winter tourism (the nearest ski area is Kranjska Gora) faces substantial challenges. A marked decline in days with snow cover (Fig. 8) is projected across most scenarios and altitude levels, jeopardizing the viability of winter sports. While the RCP2.6 scenario offers a glimmer of hope with the potential for increased snowfall at higher elevations, realizing this outcome necessitates robust mitigation efforts.

**Fig. 9:**
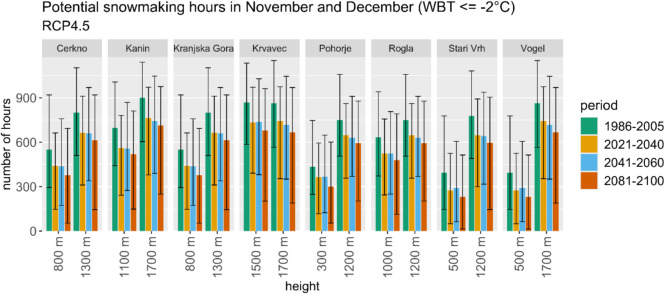
Representation of CDS data and projections of the average number of potential snowmaking hours in November and December for various ski areas in Slovenia at two heights for the reference period and three future periods according to RCP4.5 scenarios

The sustainability of winter tourism is further threatened by the projected decrease in potential snowmaking hours under RCP4.5 (Fig. 9) and RCP8.5, while RCP2.6 presents a more stable outlook. The complex interaction between increased temperatures and altered precipitation patterns underscores the vulnerability of Rateče to climate change.

## Discussion

The Mediterranean basin is increasingly grappling with the intensifying impacts of climate change, which pose significant challenges to its tourism industry. The core of these challenges is the escalating frequency and severity of heatwaves (Amelung and Viner 2006). We have also shown in the example for Bilje that even though the *CIT: 3S* index is showing the trend and projections of increasing ideal summer conditions, this needs to be considered with caution due to the observed and projected increase in hot days and tropical nights. For the Croatian island of Lošinj, the TCI projections showed a decrease in unacceptable conditions and an increase in acceptable and ideal conditions (Čavlek et al. 2019). More than 20 years ago, researchers wrote how some parts of the Mediterranean are already experiencing peak-season temperatures that tourists find unbearable (Morgan et al. 2000). As early as 2003, experts sounded the alarm about rising temperatures by 0.3 to 0.7 °C per decade, and increasing number of days with a maximum daily temperature over 40 °C (Todd 2003). This not only diminishes visitor comfort but also poses serious health risks, particularly for vulnerable populations. As our research shows, also Bafaluy et al. (2014) suggested a focus on spring and autumn tourism, which could spread visitor flow throughout the year. However, analysing the data on a monthly scale, we found differences between activities. Further projections painted a grim picture: by 2080, the Mediterranean could be too scorching for those seeking summer getaways being too hot in the summer, making northern Europe more attractive (Amelung and Viner 2006). Climate change is also altering the region's precipitation patterns. We have described a projected decrease in days with at least 1 mm of precipitation for summer months, increasing the frequency or length of droughts. Todd (2003) also projected a decrease in the amount of summer precipitation by 15%. On the other hand, an increase in heavy precipitation events in early summer and autumn is projected in Slovenia. The water supply is also likely to become an increasingly contentious issue in the Mediterranean, particularly between the local residents and the providers of tourist attractions such as golf courses and swimming pools. Many providers will likely face a rise in the cost of insurance due to the increasing frequency and severity of extreme weather events (Nicholls 2006). The adaptation measures advised for the Mediterranean range from technical solutions (e.g. air-conditioning, water-savings, shading and water-based cooling) (e.g. Nicholls 2006; Bilgin et al. 2024) to individual and organisational intra-day adaptation to the summer heat and tourism offer diversification in response to the prolongation of the season (Čavlek et al. 2019; eco-union 2019; Cardell et al. 2022).

Research on the impact of climate change on tourism is limited in the Pannonian Basin, but a notable exception is a study by Kovács and Király (2021) on *TCI* projections for Hungary. Their findings indicate that optimal conditions for tourism occur during spring and autumn, while summer experiences a decline in climate potential. Our research in the subcontinental region of Slovenia, although using different indices, aligns with these observations in the form of derived impacts. Similar to the Hungarian case study, Novo Mesto demonstrates the potential to extend the outdoor tourist season through improved climate conditions in spring and autumn. This is crucial for adapting to changing climatic circumstances. Both Slovenia and Hungary face challenges due to increasing heatwaves and extreme weather events during summer. Elevated temperatures strain tourism and community infrastructure by increasing energy consumption for cooling, while extreme weather events pose significant risks to infrastructure, tourist safety, and local communities.

Climate change is dramatically altering high-altitude environments. In the European Alps, this is evident from many researches on declining snow cover, melting permafrost, and glacier retreat (e.g. Bausch 2019; Malasevska et al. 2020; Salim et al. 2021; Vij et al. 2021). Ideal mountaineering conditions are becoming less predictable and shifting towards earlier or later seasons. Conversely, lower altitudes may benefit from climate change. Finger and Lehmann (2012) project increased outdoor recreation visits in Switzerland due to rising temperatures and reduced late-summer precipitation, particularly in August and September. Rudel et al. (2007) predict extended thermal comfort periods in Austria, though heat stress will increase in some regions. Pröbstl-Haider et al. (2015, 2021) identify both opportunities and challenges for Austrian tourism. While increased temperatures may boost lake tourism and extend seasons for activities like hiking and biking, they can also negatively impact climbing, fishing, and water sports. Tourist preferences for sunshine vary, with relaxation-oriented visitors more positive than activity-oriented ones. Austrian data is valuable for Slovenia, especially for the Alpine region, and we can see similar trends in more ideal summer conditions in the mountains and potential problems with the winter season due to decreased snow cover and warmer conditions. Snowmaking, securing tourism safety and product diversification (both within winter and to other seasons) have been the most common proposed adaptation measures for ski tourism (e.g. Berard-Chenu et al. 2022; Pantić et al. 2025).

To ensure the long-term resilience of the tourism industry, a comprehensive adaptation strategy in Slovenia is imperative. This should encompass measures to capitalize on potential growth in summer tourism while simultaneously investing in snowmaking infrastructure and developing alternative winter activities to mitigate the impacts of reduced snow cover. Continuous monitoring and evaluation of climate projections are essential to inform adaptive decision-making and minimize the overall vulnerability of the region to climate change. Future research needs to focus on the effectiveness and feasibility of adaptation measures. There is limited data on the feasibility and effectiveness of tourism climate adaptation measures beyond technical solutions such as snowmaking and air-conditioning. While the IPCC Sixth Assessment Report (IPCC 2022) included feasibility and effectiveness analysis for other areas, this was not done for tourism. Owen (2020) analysed 2294 articles on climate adaptation effectiveness. She found only 110 cases that analysed actual implementation and included 918 adaptation activities. Of these 918, no adaptation activity was tourism-specific. This reflects the difficulty of assessing the impact of more complex social adaptations that go beyond mere technical solutions. For example, while tourism diversification is a common adaptation recommendation, to our knowledge, there is no research on the effectiveness of diversification as a climate change adaptation measure. We expect this type of research to develop with the growth of adaptation implementation initiatives.

Indicators offer a simplified representation of intricate climate processes, providing valuable overviews while also carrying risks of misinterpretation and oversimplification (Scott et al. 2016). Their development and implementation necessitate a collaborative, transdisciplinary approach (Strasser et al. 2019). Effective indicator usage hinges on robust stakeholder engagement, recognizing that indicator interpretation is a co-constructed process influenced by specific contexts. Diverse stakeholder perspectives highlight the importance of inclusive engagement. The non-linear nature of both climate systems and tourism responses complicates impact assessments. Climate systems exhibit non-linear dynamics, with critical thresholds capable of triggering disproportionate impacts across regions. Tourism is similarly complex, influenced by factors like perceived temperature, which may not directly correlate with actual temperature but significantly affects visitor behavior (Arabadzhyan et al. 2021). The importance of national basic climate variables use was shown especially in the case of *HCI: Urban* showing almost only ideal days in the summer, but the increasing number of hot days setting a warning of possible high level of heat stress.

CDS provides a comprehensive dataset of climate indicators and projections relevant to the tourism sector. However, several caveats must be considered. Firstly, the complexity of climate systems necessitates caution to avoid oversimplification or misinterpretation of indicators, as described above. Secondly, discrepancies between CDS data and national ARSO data, particularly evident in the *HCI: Urban* reference period, highlight potential biases. Absolute values should, therefore, be interpreted with prudence, and the utilization of projected deviations is recommended. Climate projections are indispensable for assessing future tourism impacts. Ensemble modeling aids in quantifying uncertainties inherent in such projections. Nevertheless, the higher uncertainty associated with CDS projections, especially for snow indicators, diminishes the reliability of median values for representing the impact in the tourism sector.

## Conclusions

This study analyzed the influence of climate change on tourism across three distinct climatic regions in Slovenia: sub-Mediterranean, subcontinental, and subalpine. The findings reveal a complex interplay of opportunities and challenges for the tourism sector. The sub-Mediterranean region is experiencing an extended tourism season, primarily due to warmer spring temperatures. While summer conditions remain favorable for water-based tourism, a projected increase in hot days and tropical nights poses challenges. A decline in summer precipitation is expected, potentially impacting outdoor activities. However, an increase in heavy precipitation events in autumn highlights the need for effective flood management. The subcontinental region benefits from favorable climate conditions for urban tourism throughout the year. Despite favourable overall conditions, a projected increase in hot days during summer underscores the need for adaptation measures to maintain thermal comfort. While summer remains the peak season, the extension of suitable conditions into spring and autumn offers opportunities for tourism diversification. The subalpine region faces significant challenges due to declining snow cover and reduced snowmaking potential, jeopardizing winter tourism. While summer conditions are projected to improve, the region's economic reliance on winter tourism necessitates adaptation strategies, including diversification of activities.

The study highlights the importance of considering both average climate conditions and extreme weather events. While European datasets offer valuable insights, their limitations, such as lack of bias correction, necessitate cautious interpretation, primarily focusing on deviations rather than absolute values. The database might also cease to be maintained or accessible (the recent case of the *CIT: 3S* and *HCI: Urban* scores), therefore, the use of national meteorological datasets is crucial for accurate and reliable input for the calculations of scores. It is important to continue monitoring and evaluating climate trends and their implications for the tourism sector, leveraging both national and European datasets. This study can be used as an example to other countries or destinations on how to use different datasets with appropriate interpretation. Relying solely on tourism climate indices may be misleading, as their exclusive use can lead to an incomplete understanding of the underlying climate dynamics. Therefore, we recommend including basic meteorological variables in the analysis.

Effective adaptation strategies are crucial for mitigating the negative impacts of climate change on tourism and capitalizing on emerging opportunities. Further research is needed to explore the indirect impacts of climate change on tourism, such as changes in consumer behavior and competitive dynamics. We recommend developing region-specific adaptation plans to address the unique challenges and opportunities presented by climate change, promoting tourism diversification to reduce reliance on climate-sensitive activities.

## Data Availability

The datasets generated during and/or analysed during the current study are available from the corresponding author on reasonable request.
